# Life Expectancy in a Large Cohort of Type 2 Diabetes Patients Treated in Primary Care (ZODIAC-10)

**DOI:** 10.1371/journal.pone.0006817

**Published:** 2009-08-28

**Authors:** Helen L. Lutgers, Esther G. Gerrits, Wim J. Sluiter, Lielith J. Ubink-Veltmaat, Gijs W. D. Landman, Thera P. Links, Reinold O. B. Gans, Andries J. Smit, Henk J. G. Bilo

**Affiliations:** 1 Department of Internal Medicine, University Medical Center Groningen, Groningen, the Netherlands; 2 Diabetes Center, Isala Clinics, Zwolle, the Netherlands; 3 Department of Endocrinology, University Medical Center Groningen, Groningen, the Netherlands; 4 Family practice't Veen, Hattem, the Netherlands; 5 Department of Medicine, University of Groningen, Groningen, the Netherlands; Institute of Preventive Medicine, Copenhagen University Hospital, Denmark

## Abstract

**Background:**

Most longitudinal studies showed increased relative mortality in individuals with type 2 diabetes mellitus until now. As a result of major changes in treatment regimes over the past years, with more stringent goals for metabolic control and cardiovascular risk management, improvement of life expectancy should be expected. In our study, we aimed to assess present-day life expectancy of type 2 diabetes patients in an ongoing cohort study.

**Methodology and Principal Findings:**

We included 973 primary care type 2 diabetes patients in a prospective cohort study, who were all participating in a shared care project in The Netherlands. Vital status was assessed from May 2001 till May 2007. Main outcome measurement was life expectancy assessed by transforming actual survival time to standardised survival time allowing adjustment for the baseline mortality rate of the general population. At baseline, mean age was 66 years, mean HbA_1c_ 7.0%. During a median follow-up of 5.4 years, 165 patients died (78 from cardiovascular causes), and 17 patients were lost to follow-up. There were no differences in life expectancy in subjects with type 2 diabetes compared to life expectancy in the general population. In multivariate Cox regression analyses, concentrating on the endpoints ‘all-cause’ and cardiovascular mortality, a history of cardiovascular disease: hazard ratio (HR) 1.71 (95% confidence interval (CI) 1.23–2.37), and HR 2.59 (95% CI 1.56–4.28); and albuminuria: HR 1.72 (95% CI 1.26–2.35), and HR 1.83 (95% CI 1.17–2.89), respectively, were significant predictors, whereas smoking, HbA_1c_, systolic blood pressure and diabetes duration were not.

**Conclusions:**

This study shows a normal life expectancy in a cohort of subjects with type 2 diabetes patients in primary care when compared to the general population. A history of cardiovascular disease and albuminuria, however, increased the risk of a reduction of life expectancy. These results show that, in a shared care environment, a normal life expectancy is achievable in type 2 diabetes patients.

## Introduction

The incidence and prevalence of diabetes mellitus has risen worldwide during the past few decades. Recently published data from the Framingham Heart Study showed an absolute increase in the incidence of diabetes of ∼2.5% yearly during the 1990s compared to the 1970s [1]. The proportion of cardiovascular disease attributable to diabetes mellitus has increased as well [2]. Other studies over the last decades of the previous century also showed higher mortality rates in diabetes mellitus compared to the general population, mostly due to cardiovascular events [3]–[7]. However, since the progress in effective pharmaceutical interventions and more stringent regimens for the treatment of hyperglycemia, hypertension, dyslipidemia, and other cardiovascular risk factors, trends towards a reduction of (cardiovascular) mortality rates amongst diabetic patients have been reported [8]–[12]. This improvement of survival was hoped to eventually be comparable to the decrease in cardiovascular mortality rates in the general population thanks to aggressive management of cardiovascular risk factors.

Most published data were extracted from representative national cohorts in North-America or the United Kingdom. Some reports showed a decline in mortality rates amongst diabetic men only, whereas women showed an increase or no change in mortality rates at all [4], [8], [11], [12]. A recently published Scandinavian study showed a substantial decrease in mortality rates from coronary heart disease in all age groups irrespective of sex and diabetes status over two consecutive time periods. However, the more than twofold higher mortality from coronary heart disease in diabetes patients compared to the non-diabetic population remained over time. Still, these findings suggested a longer survival in diabetes patients resulting from intensified treatment of cardiovascular risk factors [13].

Our aim was to investigate present-day life expectancy in a type 2 diabetes population treated in primary care, with additional support both for patients and health care professionals in a shared care setting in a Western European country.

## Methods

### Participants

**Figure 1:**
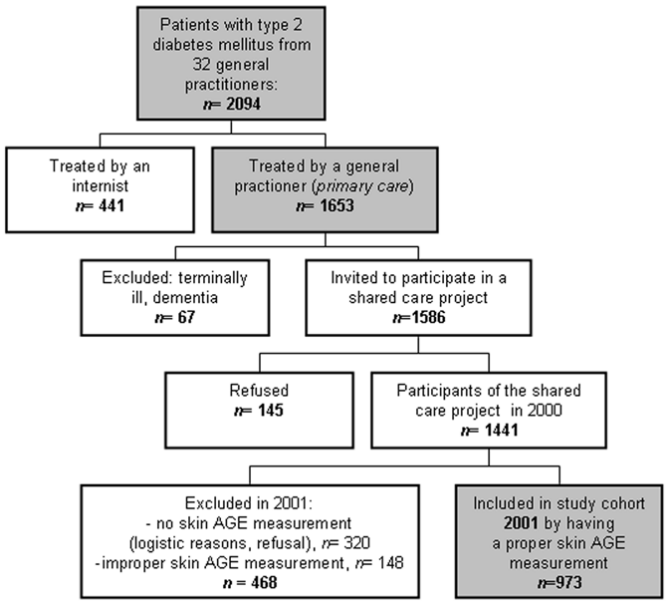
Flowchart of the enrolment of the type 2 diabetes study cohort from 32 general practitioners of a district in The Netherlands.

In 2001, 973 type 2 diabetes patients participated in a cross-sectional study with measurements of skin advanced glycation endproduct (AGE) accumulation as described previously [14]. This study was embedded in a long-term shared care project (ZODIAC: Zwolle Outpatient Diabetes project Integrating Available Care) concerning a primary care treated population-based sample of type 2 diabetes patients in an eastern district of The Netherlands. Figure 1 shows an overview of the enrolment of the current study cohort started from the beginning of the ZODIAC. During this project, 32 general practitioners (GPs) were supported by hospital diabetes specialist nurses and consultant-physicians [15]. In short, all type 2 diabetes patients were exclusively treated by their GPs and visited the diabetes specialist nurses for evaluation of metabolic control and diabetes related complications annually. After these evaluations, treatment advice for individual patients as well as for benchmarking was given to the GPs by internists in the Isala Clinics in Zwolle, the Netherlands. Advices and referrals were based on guidelines of the Dutch College of General Practitioners [16].

Patients with a cognitive disability or terminal disease were not included in the ZODIAC study because of their inability to undergo educational programs. Furthermore, patients who were physically unable to visit the diabetes specialist nurse at the outpatient clinic were not enclosed in the present cohort.

### Ethics Statement

This study was approved by the local ethical committee of the Isala Clinics, Zwolle, The Netherlands and all patients gave written informed consent.

### Description of Procedures

Methods of clinical data collection and laboratory assessments have been described in detail elsewhere [14]. Before participation in our study, based on the 1997 ADA guidelines and the Dutch primary care standard [16], diagnosis of diabetes mellitus was already made in individuals with fasting plasma glucose levels ≥7.0 mmol/liter and the following definitions representing diabetic complications at baseline were: retinopathy, which was defined as the presence of at least background retinopathy or a history of laser coagulation for diabetic retinopathy. Albuminuria was defined as an albumin-to-creatinine ratio >3.5 mg/mmol for women or >2.5 mg/mmol for men. Diminished sensibility at least at one foot was considered as neuropathy, tested with a 5.07 Semmes-Weinstein monofilament, applied on three areas of each foot. The presence of microvascular disease was defined as meeting the criteria of retinopathy, albuminuria, and/or neuropathy. The presence of cardiovascular disease at baseline was defined when meeting at least one aspect of cardiovascular disease: ischemic heart disease (IHD), International Classification of Diseases ninth revision (ICD-9), codes 410–414 and/or a history of coronary artery bypass surgery or percutaneous coronary intervention, cerebrovascular accidents including transient ischemic attacks (CVAs/TIAs) and/or peripheral vascular disease (PVD). PVD was defined as surgical intervention, history of claudication and/or absent pulsations of ankle or foot arteries (absence of pulsations of the dorsalis pedis arteries bilaterally was not scored as PVD when tibial posterior artery pulsations were present).

Mortality was registered from the date of inclusion until May 2007. Death was certified according to the following procedure. In addition to the list of deceased patients reported in the files of the scheduled annual follow-up visits, survival status of the patients was obtained from the local hospital information system and verified with the GPs. Date of death was collected likewise. None of the GPs had involvement or interest in study outcome. Causes of death were coded according to ICD–9 and categorised as: neoplasms (140–239), diseases of the cardiovascular system (390–459), diseases of the respiratory system (460–519), diseases of the digestive system (520–579), diseases of the genitourinary system (580–629), injury and poisoning (800–999). Sudden death, with symptoms present for less than one hour, was encoded in the category of coronary heart disease. For the in-hospital deaths, the medical records were retrieved. For the out-of-hospital deceased patients, the assigned causes of death by the GPs were obtained from the medical records of the GPs. The coded causes of death were combined to all-cause mortality (all codes) and cardiovascular mortality (390–459 or sudden death).

### Statistical Methods

Life expectancy analysis was performed primarily by the use of ‘standardised survival time’ (SST), which is a novel approach to survival analysis [17]. SST is another expression of follow-up time than survival time in years. This method provides survival time, which is adjusted for the median residual life span of individuals in the general population with the same age and sex. Due to this standardisation of survival time there is no influence of the interactions between age and the presence or effects of other risk factors due to age. Furthermore, it allows assessment of the effectiveness of treatment on regaining a normal residual life span. SST was calculated as the ratio between the observed survival time (follow-up time) of an individual and the median residual life span of individuals with the same age and sex in the general population at the starting date of the study. The median residual life span was derived from gender specific reports provided by the Dutch Central Office of Statistics, which is the national Dutch institution of statistics and demographics [18]. The SST at baseline is defined as 0, and a SST ratio of 1 is defined as follow-up time corresponding with the life expectancy of the general population. Direct comparisons between study samples and the general population were done by comparing the 95% confidence interval (CI) of each median SST with an expected value of 1. The 95% CI of mortality at a SST of 0.25 and 0.5 were calculated and compared with the expected mortality as calculated for the age and gender matched general population, assuming Poisson distribution of the events. Kaplan-Meier curves were constructed for survival and for standardised survival. A Cox proportional hazard model to estimate hazard ratios (HR) and 95% CI was used in the standard way using survival time in years, and additionally by using SST. Methodologically, it is allowed to use SST instead of survival time in years in a Cox-regression model, as it is consistent with the preconditions of a Cox regression analysis: an increase in mortality and an increase in follow-up time have to be present. This new statistical approach underlines the prognostic value of the mentioned risk factors, irrespective of age and sex. Eliminating the effects of age and sex excludes the influence of disease-specific risk of age and sex in the standardised analysis. P values <0.05 were considered to be statistically significant. Clinical and laboratory variables with an expected effect on mortality risk were first analysed in a univariate analysis, and secondly, in a multivariate model. Detailed analyses were performed specifically for two end-points: all-cause mortality and cardiovascular mortality.

## Results

Characteristics of the 973 type 2 diabetes patients at baseline (2001) are shown in Table 1. The population had a relatively short median diabetes duration of 4.2 (interquartile range 1.6–8.3) years and on average an acceptable to good glycemic control (mean HbA_1c_ 7.0%). Table 1 also shows the baseline characteristics of patients when subdivided in survivors (791 patients) and non-survivors. At the end of a median follow-up duration of 5.4 years (interquartile range 5.1–5.6), 165 patients had died (17%); 17 patients were lost to follow-up. Minimum follow-up duration of all survivors was 5.0 years. Ten of the lost to follow-up persons had a last visit to the outpatient clinic between baseline and the end of follow-up; this last registered visit date was defined as the end of follow-up for these patients. The remaining 7 persons lost to follow-up had a mean age of 68 years, were non-smokers, and 2 patients had cardiovascular disease at baseline. Their median diabetes duration was 9.7 years with a mean HbA_1c_ of 7.5%.

**Table 1:** Baseline characteristics of type 2 diabetes patients: total and subdivided in survivors and non-survivors expressed as mean±SD or *n* (%).

Characteristic	Total (N = 973)	Survivors (N = 791)	Non-survivors (N = 165)	p-value
Age in years	66.4 (11.3)	64.8 (11.1)	73.6 (9.4)	<0.001
Male gender (%)	47	46.3	50.9	0.278
Smoking (%)	19.3	19.3	20.6	0.71
Body mass index in kg/m^2^	29.38 (4.87)	29.5 (4.8)	28.7 (5.1)	0.077
Systolic blood pressure in mmHg	146.01 (20.15)	145 (20)	149 (20)	0.015
Diastolic blood pressure in mmHg	81.18 (10.34)	81 (10)	80 (11)	0.046
Diabetes duration in years	a4.16 (1.62–8.31)	a3.92 (1.50–8.04)	a5.03 (2.40–10.8)	0.002
HbA_1c_ (%)	6.96 (1.3)	6.95 (1.32)	6.998 (1.23)	0.69
Creatinine in µmol/l	96.0 (19.88)	94.56 (17.6)	103.16 (27.69)	<0.001
Creatinine clearance in ml/min	76.13 (26.91)	78.8 (26.6)	63.22 (24.75)	<0.001
Urinary albumin-to-creatinine ratio in mg/mmol	a1.49 (0.80–4.17)	a1.35 (0.75–3.44)	a3.09 (1.23–11.01)	0.001
Total cholesterol in mmol/l.	5.16 (1.01)	5.17 (1.02)	5.08 (1.00)	0.302
Cholesterol-to-HDL ratio	4.34 (1.23)	4.37 (1.21)	4.22 (1.36)	0.171
HDL cholesterol in mmol/l	1.25 (0.33)	1.24 (0.32)	1.29 (0.35)	0.141
LDL cholesterol in mmol/l	2.87 (0.93)	2.85 (0.92)	2.92 (0.98)	0.388
Triglycerides in mmol/l	2.32 (1.36)	2.39 (1.40)	2.03 (1.14)	0.002
Microvascular disease (%)	54.1	50.2	70.9	<0.001
Retinopathy (%)	19.6	18.5	24.8	0.050
Microalbuminuria (%)	25.5	21.4	45.5	<0.001
Neuropathy (%)	29.1	26	40.6	<0.001
Cardiovascular disease (%)	39.5	34.6	63.6	<0.001
Ischemic heart disease (%)	21.5	19.6	30.3	0.002
Cerebrovascular disease (%)	7.8	6.4	14.5	<0.001
Peripheral vascular disease (%)	23.0	18.1	47.3	<0.001
RAS-inhibitors^b^ (%)	37.2	36.5	39.4	0.489
Lipid-lowering drugs^c^ (%)	29.8	30.7	26.1	0.234
Antiplatelet drugs (%)	24.9	22.1	38.2	<0.001
Diabetes treatment – Diet only (%)	20.2	21.4	16.4	
Oral medication (%)	64.1	64.3	63	
Insulin (%)	9.8	8.3	15.8	
Both (%)	5.9	5.9	4.8	

Seven patients were lost to follow-up and did not define the baseline characteristics of the survivors/non-survivors.

a Median and interquartile range.

b Angiotensin-converting enzyme inhibitors and Angiotensin II receptor blockers.

c Large majority represented by statins (99%). Reference values of the laboratory: HbA_1c_ 4.0–6.0%, creatinine 70–110 µmol/l, creatinine clearance (Cockcroft-formula) 80–120 ml/min, urinary albumin-to-creatinine ratio 0–2.5 for men and 0–3.5 for women, total cholesterol 3.5–5.0 mmol/l.

The proportion of prescribed lipid-lowering drugs, renin-angiotensin system (RAS) inhibitors and antiplatelet therapy at baseline, is shown at the end of Table 1. At baseline, antiplatelet drugs were significantly more prescribed in the non-survivors compared to the survivors. The proportion of cardiovascular deaths in the study population (47%) was increased compared to the general population. In 2007, 31% of all deaths in the general Dutch population were due to cardiovascular disease, with a highest relative incidence of 38% cardiovascular deaths in the population above 85 years [18].

**Figure 2:**
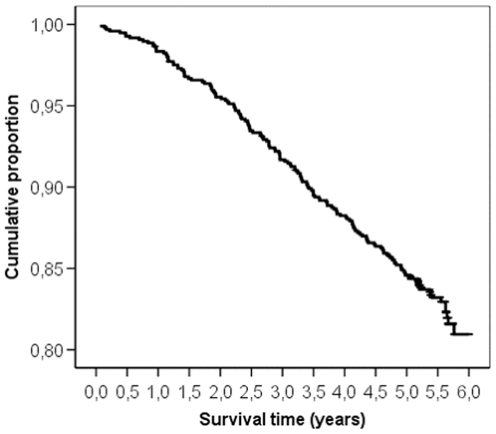
Kaplan-Meier survival curve for survival in years in the entire type 2 diabetes group.

**Figure 3:**
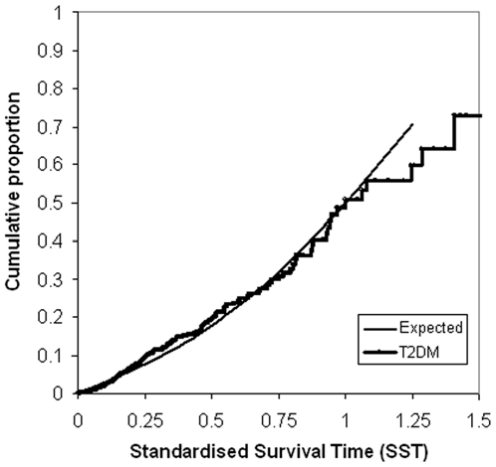
Kaplan-Meier plot of the cumulative proportions of deaths against Standardised Survival Time. Survival curve for survival expressed as Standardised Survival Time (SST) in the entire type 2 diabetes group. The median SST in type 2 diabetes mellitus (T2DM) 1.00 is not different from the general Dutch population 1.00 (Expected); the observed mortality (all-cause) at SST 0.25 and 0.50 of 0.09, respectively 0.20 does not significantly differ from the general Dutch population (0.08 respectively 0.18, p>0.1).

Figure 2 shows the Kaplan-Meier curve of the cumulative proportion of survivors in our type 2 diabetes population against survival time in years. A Kaplan-Meier plot of the cumulative proportion of deaths in our study population against standardised survival time is shown in figure 3; the expected mortality for the age- and gender-matched general population is also shown. The median standardised survival time in our study population was 1.00 [95% confidence interval (CI) 0.88–1.12] and did not differ from the general population. The cumulative proportion of deaths at half standardised survival time (SST = 0.50) was 0.20 (95% CI 0.16–0.23), which again did not differ from the expected value of 0.18 in the general population.

**Figure 4:**
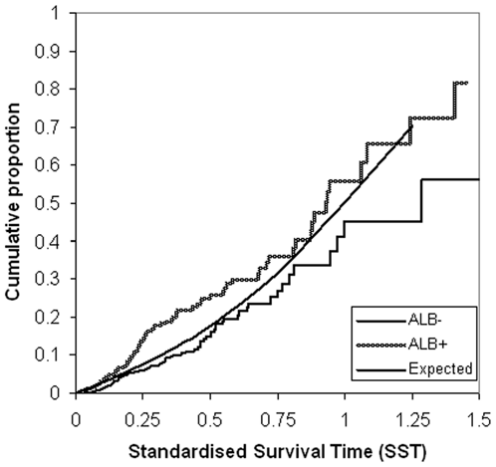
Kaplan-Meier plot of the cumulative proportions of deaths in patients with albuminuria against Standardised Survival Time. Cumulative proportions of deaths (all causes) against Standardised Survival Time (SST) in type 2 diabetes patients with albuminuria (Alb) yes/no (+/−), compared to the expected deaths of the general population. Differences in mortality between the type 2 diabetes-subgroups and the general population are tested at SST = 0.25 and SST = 0.5. Mortality rate at SST 0.25 is 0.15 [95% confidence interval (CI) 0.10–0.19] and the expected value is 0.076 (p = 0.002). At SST 0.50 mortality rate is 0.26 (95% CI 0.19–0.33), which was also higher than the expected value of 0.18 (p = 0.014).

**Figure 5:**
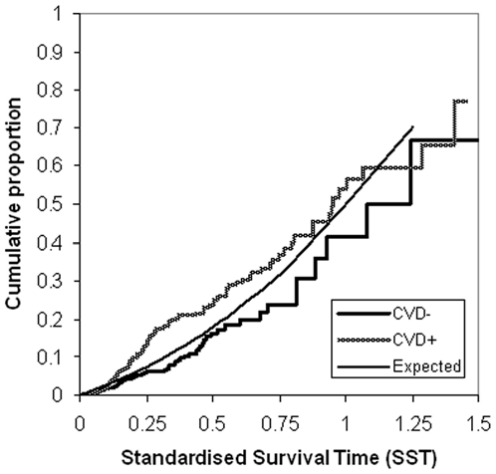
Kaplan-Meier plot of the cumulative proportions of deaths in patients with previous cardiovascular disease against Standardised Survival Time. Cumulative proportions of deaths (all causes) against Standardised Survival Time (SST) in type 2 diabetes patients with previous cardiovascular disease (CVD) yes/no (+/−), compared to the expected deaths of the general population. Differences in mortality between the type 2 diabetes-subgroups and the general population are tested at SST = 0.25 and SST = 0.5. Mortality rate at SST 0.25 is 0.13 (95% CI 0.096–0.17) and an expected value is 0.076, p<0.001. At SST 0.50 mortality rate is 0.25 (95% CI 0.19–0.30) and the expected value is 0.18, p<0.0001.

Figure 4 shows the mortality rate in type 2 diabetes patients with albuminuria at SST 0.25 of 0.15 (95% CI 0.10–0.19), which was higher than the expected value of 0.076 (p = 0.002). At SST 0.50 mortality rate was 0.26 (95% CI 0.19–0.33), which was also higher than the expected value of 0.18 (p = 0.014). This also proved to be the case for type 2 diabetes patients with a history of cardiovascular disease, who had a higher mortality at SST 0.25 [0.13 (95% CI 0.096–0.17), p<0.001] and at SST 0.50 [0.25 (95% CI 0.19–0.30), p<0.0001], figure 5.

Table 2 shows the hazard ratios (HRs) and 95% CI of univariate and multivariate Cox-regression analyses for all-cause mortality. The HRs in the univariate analyses are higher for all cardiovascular disease items compared to the method of using SST in the model. In the multivariate analysis, predictive factors for all-cause mortality were comparable for both methods when age and gender were included in the model of the standard method: a history of cardiovascular disease (HR 1.79 and 1.71) and, albuminuria (HR 1.79 and 1.72). Univariate analysis of the endpoint: cardiovascular mortality (not shown in Table 2) resulted in the same significant predictive factors, but with higher HRs. Multivariate analysis of cardiovascular mortality resulted in the same significant predictive factors with higher HRs (SST) as well: albuminuria 1.83 (95% CI 1.17–2.89); history of cardiovascular disease 2.59 (95% CI 1.56–4.28). Smoking, systolic blood pressure, diabetes duration and HbA_1c_ did not reach significance in both multivariate models.

**Table 2:** Predictors of overall mortality in type 2 diabetes mellitus by univariate and multivariate Cox regression analysis using “survival in years” ( = standard method) and using “standardised survival time”.

Predictors of all-cause mortality	Survival in years			Standardised survival time^a^			
	Univariate						
	HR	95% CI	*p-value*		HR	95% CI	*p-value*
Gender (man = reference)	0.84	0.62–1.14	0.25		0.91	0.67–1.24	0.56
Age	1.08	1.06–1.10	<0.001		1.00	0.98–1.02	0.90
Smoking	1.10	0.75–1.60	0.63		1.49	1.02–2.18	0.039
Systolic blood pressure	1.01	1.00–1.02	0.016		1.00	0.99–1.01	0.66
Diabetes duration	1.03	1.02–1.05	0.001		1.02	1.00–1.04	0.073
HbA_1c_	1.02	0.91–1.15	0.71		1.08	0.95–1.22	0.23
Albuminuria (yes/no)	2.33	1.71–3.17	<0.001		1.81	1.32–2.46	<0.001
History of cardiovascular disease (yes/no)	2.95	2.15–4.06	<0.001		1.87	1.35–2.58	<0.001
Use of lipid-lowering drugs (yes/no)	0.83	0.59–1.18	0.30		1.34	0.80–1.62	0.48
Use of antiplatelet drugs (yes/no)	2.00	1.46–2.74	<0.001		1.47	1.07–2.01	0.018

Multivariate						
Gender (man = reference)			NS	x	x	x
Age	1.07	1.05–1.09	<0.001	x	x	x
Smoking (yes/no)			NS			NS
Systolic bloodpressure			NS			NS
Diabetes duration			NS			NS
HbA_1c_			NS			NS
Albuminuria (yes/no)	1.79	1.30–2.46	<0.001	1.72	1.26–2.35	0.001
History of cardiovascular disease (yes/no)	1.79	1.29–2.50	0.001	1.71	1.23–2.37	0.001
Use of lipid-lowering drugs (yes/no)			NS			NS
Use of antiplatelet drugs (yes/no)			NS			NS

Abbreviations: HR, hazard ratio; CI, confidence interval; NS, not significant.

a The standardised survival time was calculated as the ratio between the observed survival time of an individual and the median residual life span of individuals with the same age in the general population.

## Discussion

This study shows a normal median overall life expectancy in a defined cohort of type 2 diabetes patients treated in a primary care setting, during a follow-up period from 2001 till 2007. This finding strongly suggests that current available treatment strategies may eventually lead to a life expectancy equal to the general population in this subset of type 2 diabetes patients. Secondly, in this type 2 diabetes study population, patients with a history of cardiovascular disease and/or the presence of albuminuria still had an increased risk to die before their median life expectancy was reached. The differences in effects of all items of cardiovascular disease on ‘survival in years’ and SST, could be explained by the age correction enclosed in the SST – method. As the prevalence of cardiovascular diseases is increasing with increasing age, SST does have definite advantages compared to the ‘classical survival time’ in identifying premature mortality.

Finally, we still found an increased proportion of deaths due to cardiovascular disease compared to the general population (47% versus 31%). This is in agreement with established observations of increased cardiovascular disease in diabetes, and also with the fact that the presence of classical cardiovascular risk factors still is most intimately related to life expectancy reduction [13], [19].

The United Kingdom Prospective Diabetes Study reported a 5 years reduction of life expectancy for males aged 45 to 50 years at diagnosis of diabetes when compared to the general United Kingdom population [6]. Estimations of reduction of life expectancy for patients with diabetes diagnosed at an older age are not presented explicitly in this paper, but might be smaller than 5 years, as other studies showed that reduction of life expectancy decreases with diagnosis at older age [5], [12], [20].

A large study of the non institutionalised United States population, which was conducted between 1971 and 1993, showed a median reduced life expectancy of 8 years for the diabetic population aged 55–64 years, and a 4 years reduction for the diabetic population aged 65–74 years [4]. However, these studies were all executed in a period during which treatment with statins, angiotensin-converting enzyme inhibitors and angiotensin-1 receptor blockers, and antiplatelet medication was much less common practice. A more recent study, showing slightly increased mortality in women but no excess mortality in men, included exclusively patients diagnosed with type 2 diabetes mellitus over the age of 65 [12]. Our study is of additional value, as we included primary care type 2 diabetes patients of all ages, representing a large amount of the type 2 diabetes patients in The Netherlands, where the majority of subjects with type 2 diabetes is treated in primary care according to national guidelines. Sixty-four percent of our study population was diagnosed with type 2 diabetes mellitus before the age of 65 years.

A previous study in the first ZODIAC-cohort (1998) reported an annual mortality rate of 4.8% between 1998 and 2000 (the first three years of the shared care project), definitely higher than the mortality rate in the present analysis (∼3%), which was performed over the subsequent years within this shared care environment [21]. This difference could be explained by the fact that the earlier analysis was performed in a more extended type 2 diabetes cohort, which also included patients who were referred to secondary care. It is also possible that the cohort as presented in this first analysis had yet to benefit from longer term participation in a shared-care environment with supportive care and monitoring of implementation of the guidelines.

More than half of our population received either a statin, RAS-inhibitor or aspirin at baseline. At follow-up, this proportion had increased to at least 80%. Widespread treatment of the traditional cardiovascular risk factors resulted in vastly improved blood pressure readings and lipid levels. This could also be the explanation for disappearance of systolic blood pressure from the model to predict mortality. Recent studies, although maybe underpowered, addressed the importance of statins and blood pressure lowering drugs in patients with type 2 diabetes mellitus, showing a reduction in cardiovascular events with these lipid-lowering drugs compared to placebo [22]–[24].

HbA_1c_ had also no effect on life expectancy in uni- and multivariate analysis. This may possibly be explained by the low number of patients with poor glycemic control (only 7% had a HbA_1c_ >9%). Alternatively, other mechanisms could be involved in the development of diabetes related complications. E.g., we recently reported increased levels of advanced glycation endproducts (AGEs) rather than HbA_1c_ in the same study group, to be related to chronic complications [14].

There are some limitations to our study regarding the possible general applicability of the results. Diabetic patients who were referred to the secondary care in the past, mainly for reasons of poor metabolic control or comorbidity, were not included in this study and almost certainly will have a reduced life expectancy. Also, there has been a selection bias by excluding diabetic patients with a very short life expectancy (terminally ill patients, cognitive disabled people and patients who were unable to undergo educational programs), as described in the methods section. Still, the selection comprised a considerable subset of the total population known with type 2 diabetes (see figure 1), and 40% of the included study population were known with cardiovascular disease at baseline.

Despite this apparent selection bias, we still are able to conclude that we defined a large subset of patients with a life expectancy comparable to that of the general population of the same age and sex. In The Netherlands, the large majority (70–80%) of type 2 diabetes patients is treated in primary care or in a shared-care setting. Therefore, this study population could be representative for the majority of type 2 diabetes patients in The Netherlands, and probably also for a larger part of type 2 diabetes patients in other countries with structured diabetes care.

Our choice to compare life expectancy of this type 2 diabetes cohort to the general population can be criticised, since the general population also includes people with diabetes, cardiovascular disease, cancer, and other life shortening diseases. We nevertheless preferred to choose the general population instead of a non-diabetic control group, since one of the aims of caregivers in medical practice is to regain a life expectancy for their patients equal to the general population when life expectancy is reduced due to a specific disease.

To visualise whether a life expectancy equal to the general population had been achieved, we used SST. Traditional survival analysis focuses more on ‘mortality’ within a certain follow-up time, but with this more conventional method it is not clear whether it is ‘normal mortality’ or ‘excess mortality’. Using SST, the mortality rate is adjusted for the median survival of subjects in the general population of the same age and sex. In this way, we eliminate the effect of age and sex, by excluding the influence of disease-specific risk of age and sex in the standardised analysis. Excess mortality or a reduced life expectancy will be identified more easily in that way. We consider the results of this study to be relevant for clinical practice, because they offer a hopeful perspective of a definitely improved life expectancy in type 2 diabetes patients. We suggest that those results are also (partly) due to the fact that these patients were and are participating in a care system promoting adherence to evidence-based guidelines and to a system emphasizing close cooperation between health care providers focusing on this patient group.

In summary, this study shows a normal life expectancy in a large subset of type 2 diabetes patients treated in a primary care setting compared to the general population. The presence of previous cardiovascular disease and albuminuria, however, is still associated with a markedly reduced life expectancy.
